# Excitation of propagating spin waves in ferromagnetic nanowires by microwave voltage-controlled magnetic anisotropy

**DOI:** 10.1038/srep25018

**Published:** 2016-04-26

**Authors:** Roman Verba, Mario Carpentieri, Giovanni Finocchio, Vasil Tiberkevich, Andrei Slavin

**Affiliations:** 1 Institute of Magnetism, National Academy of Sciences of Ukraine, Kyiv 03142, Ukraine; 2 Department of Electrical and Information Engineering, Politecnico of Bari, I-70125 Bari, Italy; 3 Department of Electronic Engineering, Industrial Chemistry and Engineering, University of Messina, I-98166 Messina, Italy; 4 Department of Physics, Oakland University, Rochester, MI 48309, USA

## Abstract

The voltage-controlled magnetic anisotropy (VCMA) effect, which manifests itself as variation of anisotropy of a thin layer of a conductive ferromagnet on a dielectric substrate under the influence of an external electric voltage, can be used for the development of novel information storage and signal processing devices with low power consumption. Here it is demonstrated by micromagnetic simulations that the application of a microwave voltage to a nanosized VCMA gate in an ultrathin ferromagnetic nanowire results in the parametric excitation of a propagating spin wave, which could serve as a carrier of information. The frequency of the excited spin wave is twice smaller than the frequency of the applied voltage while its amplitude is limited by 2 mechanisms: (i) the so-called “phase mechanism” described by the Zakharov-L’vov-Starobinets “S-theory” and (ii) the saturation mechanism associated with the nonlinear frequency shift of the excited spin wave. The developed extension of the “S-theory”, which takes into account the second limitation mechanism, allowed us to estimate theoretically the efficiency of the parametric excitation of spin waves by the VCMA effect.

Electric field control of magnetization in ferromagnets attracts a lot of attention of researchers in magnetism as it makes possible the development of novel magnetic recording and signal processing devices having low power consumption and compatible with standard CMOS technology. The electric field control could be realized using different magnetoelectric effects (see, e.g. review refs 1 and 2), among which of a particular interest is the recently discovered effect of the voltage-controlled magnetic anisotropy (VCMA)^3^,^4^,^5^. The VCMA effect manifests itself as a variation of the *perpendicular* magnetic anisotropy at the interface between a ferromagnetic metal and an insulator under the application of an interface voltage. This effect has many attractive features, including linearity (variation of the anisotropy energy is directly proportional to the applied voltage)^4^,^6^, absence of a hysteresis^7^,^8^, possibility of relatively large variations of the anisotropy field^9^,^10^ and practical absence of inertia (at least, in the gigahertz frequency range)^11^,^12^. These features make VCMA promising for various practical applications. In particular, the VCMA effect has been already proposed for use in magnetic recording^13^,^14^,^15^, control of motion of domain walls^16^,^17^,^18^ and skyrmions^19^, and for excitation of a ferromagnetic resonance^11^,^12^,^20^.

Recently, we have demonstrated analytically^21^ that by applying to a ferromagnetic metal - insulator interface a microwave voltage of a frequency *ω*_*p*_ = 2*πf*_*p*_ and a sufficient magnitude, it is possible to overcome the threshold of parametric excitation of half-frequency (*ω*_*SW*_ = *ω*_*p*_/2) short spin waves (SWs) in ferromagnetic nanowires (at the same time, the linear excitation of SWs by VCMA, when *ω*_*SW*_ = *ω*_*p*_, in a *zero bias magnetic field* is not possible^12^,^21^,^22^). This means that the VCMA effect could be promising for excitation of propagating SWs in unbiased nanosized magnetic waveguides, where these SWs can be used for microwave signal processing.

It should be noted, however, that overcoming the parametric excitation threshold does not guarantee a stable excitation of propagating SWs. In the externally driven parametric process the excitation of monochromatic half-frequency propagating SWs could be hindered by many connected parasitic nonlinear phenomena, such as modulational instability, auto-oscillations and even developed turbulence of parametrically excited SWs^23^. The exact manifestation of these nonlinear phenomena depends on the strength of the microwave driving signal, SW spectrum of a magnetic sample and peculiarities of the parametric interaction between the driving signal and parametric SWs in a given sample geometry.

The conditions necessary for a stable parametric excitation of SWs were studied in details for two- and three-dimensional geometry in the case of spatially uniform parametric pumping in the framework of the so-called L’vov-Zakharov-Starobinets “S-theory”^23^,^24^,^25^. The “S-theory” shows that the amplitudes of the excited SWs are limited by the “phase mechanism”^23^, which is the disappearance of the phase correlation between the pumping signal and the excited SWs due to the nonlinear interaction between the SWs. The “S-theory” allows one to calculate the SW amplitudes, thus providing a theoretical estimate for the parametric excitation efficiency. Unfortunately, the conclusions of the conventional “S-theory”^23^ cannot be directly translated to our case of VCMA-induced parametric excitation of SWs, where the ferromagnetic sample has to be quasi-one-dimensional (nanowire) in order to guarantee a definite direction of the SW propagation^21^, and the microwave parametric pumping is localized in the region of the VCMA gate electrode.

In our current work we study the peculiarities of the parametric excitation of SWs at a VCMA interface using the micromagnetic simulations. As it is shown below, in a real nanowire geometry the achievable magnitudes of the electric field of the microwave parametric pumping could reach the magnitudes that not only are above the threshold of parametric excitation, but are sufficient to sustain a stable generation of SWs propagating far away from the region of pumping localization and having narrow frequency linewidth. The comparison of our simulation results with the analytical results obtained using the modified “S-theory” demonstrates that in one-dimensional nanoscale ferromagnets the amplitudes of the parametrically excited SWs are limited not only by the classical “phase mechanism” (characteristic for the “S-theory” in bulk ferromagnets^23^), but, also, by an additional limiting mechanism caused by the dependence of the SW group velocity on the SW amplitude, which appears due to a non-zero nonlinear SW frequency shift.

## Results

### Micromagnetic simulations

**Figure 1:**
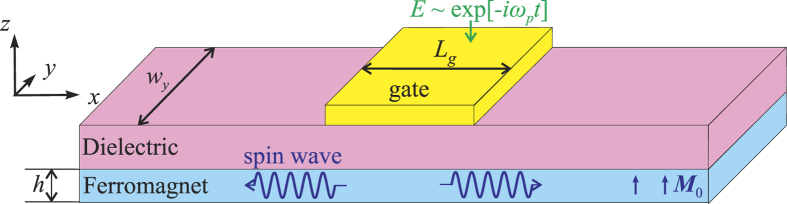
Considered system. A layout of a ferromagnetic nanowire spin wave waveguide grown on a dielectric layer and having a spatially localized conductive excitation (input) gate.

A sketch of a particular SW waveguide geometry used in our micromagnetic simulations is shown in Fig. 1. We consider a nanowire waveguide of the thickness *h* and width *w*_*y*_ made from a conductive ferromagnetic material and grown on a thin MgO dielectric layer. Note, that since VCMA is a purely interface effect^4^,^5^,^8^, the nanowire should be ultrathin (*h* ~ l nm), and, owing to the perpendicular growth anisotropy, ultrathin magnetic nanowires and films in a zero bias magnetic field, typically, exist in the static magnetic state in which the film magnetization is directed out-of-plane^26^,^27^. This magnetic configuration will be considered below.

On top of the nanowire there is a conductive excitation (or input) gate, which creates a microwave electric field *E* at the VCMA dielectric-ferromagnetic interface. This electric field drives microwave oscillation of the perpendicular interface anisotropy 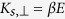 (where *β* is the magnetoelectric coefficient). The input gate covers only a part of the length *L*_*g*_ of the nanowire waveguide. In such a geometry the SWs excited at the input gate could propagate outside the input gate region, where they could be influenced by other gates performing signal processing functions and, eventually, received by an output gate.

First, we performed the simulations for a CoFeB nanowire of the thickness *h* = 0.8 nm, width *w*_*y*_ = 20 nm with a gate length of *L*_*g*_ = 40 nm (see Methods). It is known^21^, that in a narrow nanowire waveguide the excitation threshold is lower than in a wider waveguides, and, therefore, the SW parametric excitation efficiency should be higher. The magnitude of the microwave anisotropy field was fixed at Δ*B*_*a*_ = 50 mT, which corresponds to the experimentally reachable magnitude of the electric field *E* = 0.78 V/nm (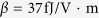)^12^, while the frequency *f*_*p*_ of this field was varied.

**Figure 2:**
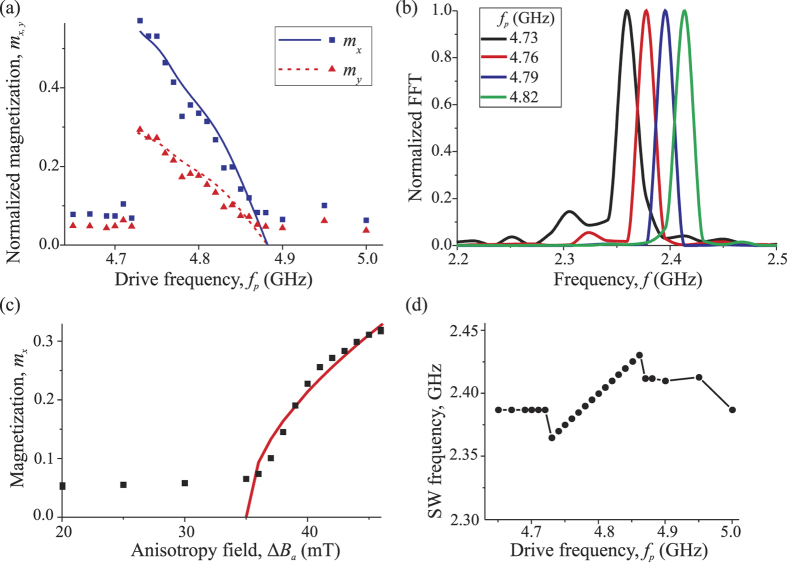
Amplitude and frequency of the excited spin waves. Normalized in-plane components 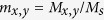 of the variable nanowire magnetization under the center of the excitation gate as functions of the pumping frequency for Δ*Β*_*a*_ = 50 mT, wire width *w*_*y*_ = 20 nm and gate length *L*_*g*_ = 40 nm (**a**) and *m*_*x*_ as a function of the pumping amplitude Δ*Β*_*a*_ at *f*_*p*_ = 4.8 GHz (**c**): symbols - micromagnetic simulations, lines - analytical theory. The frequency spectra of the micromagnetically calculated magnetization precession under the gate region and the position of their maximums as a function of the pumping frequency *f*_*p*_ are presented in the frames (**b**,**d**), respectively.

In our simulations the in-plane components of the variable nanowire magnetization (normalized by the static magnetization *M*_*s*_) were calculated under the center of the excitation gate as functions of the pumping frequency *f*_*p*_. These curves are shown in Fig. 2(a). It is clear that in the range *f*_*p*_ ∈ [4.73, 4.86] GHz the amplitude of the excited magnetization precession significantly exceeds the thermal fluctuation level. The difference between the *x* and *y* components of the variable magnetization, i.e. nonzero precession ellipticity, naturally appears due to the in-plane shape anisotropy of the nanowire, and this ellipticity remains almost constant within frequency range of parametric excitation. Frequency spectrum of the excited magnetization precession contains one dominant peak (Fig. 2(b)), the maximum of which is located *exactly* at the half of the pumping frequency *f*_*p*_ (Fig. 2(d)). This means that the microwave voltage applied to the excitation gate leads to the *parametric* excitation of SWs. Note, that the frequency distribution of excited SWs is very narrow–its width (half width at half maximum, HWHM) is equal to 9–13 MHz. This is several times smaller than in the case of SWs of the same frequency excited linearly (i.e. by the in-plane microwave field), when 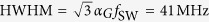. When the amplitude of the magnetization oscillations becomes larger, smaller additional peaks appear in the frequency spectrum (see curve for *f*_*p*_ = 4.73 GHz in Fig. 2(b)). However, even in that case of higher amplitude of the excited SWs the parametric excitation process remains stable, which is confirmed by the existence of only one dominant peak in the frequency spectrum of the excited SWs.

For a fixed pumping frequency the SW are excited, as expected, only above a certain threshold pumping magnitude Δ*Β*_*th*_, and above this threshold the SW amplitude increases monotonically (Fig. 2(c)). The threshold depends on the excitation frequency, mainly due to the frequency dependence of the SW group velocity (see Eq. (2)). In particular, the parametric excitation is absent for *f*_*p*_ > 4.86 GHz because at higher frequencies the threshold becomes larger then the applied pumping amplitude Δ*Β*_*a*_ = 50 mT. At the pumping frequencies below 4.73 GHz the parametric excitation is also absent, but for a different reason. For *f*_*p*_ < 4.73 GHz half of the pumping frequency lies below the bottom of the SW spectrum in the nanowire and, therefore no propagating SW can be excited parametrically in this frequency range at all.

**Figure 3:**
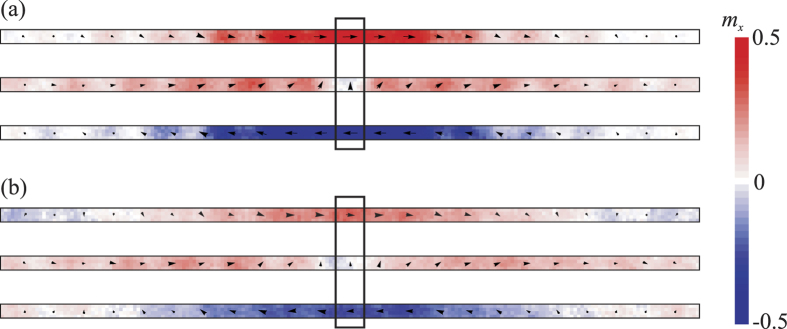
Spin wave profiles. Micromagnetic snapshots of the distributions of variable magnetization in a nanowire near the pumping gate at the moments separated by a time intervals equal to the quarter of the magnetization precession period. A part of the nanowire waveguide of the length equal to 1 μm is shown around the pumping gate, which is indicated by a black rectangle. The pumping frequency is *f*_*p*_ = 4.73 GHz (**a**) and *f*_*p*_ = 4.8 GHz (**b**), while the pumping amplitude is Δ*B*_*a*_ = 50 mT.

However, when the pumping frequency is larger than *f*_*p*_ > 4.73 GHz, the pumping excites *propagating* SWs moving along the nanowire in both directions from the excitation gate. This is clearly illustrated by Fig. 3 where three instantaneous magnetization distributions in the nanowire, corresponding to three moments separated by a quarter of a period of the magnetization precession, are presented. It is clear that the maximum of the precession amplitude moves away from the gate region and propagates for a distance that is significantly larger than the gate length. The wave number of the excited SWs depends on the excitation frequency–in the considered geometry of a nanowire with perpendicular static magnetization a higher pumping frequency *f*_*p*_ corresponds to a higher SW wave number *k*. The shortest SW excited in the considered case at *f*_*p*_ = 4.86 GHz has the wave number *k* ≈ 8 μm^−1^. The determination of the SW wave number for *f*_*p*_ = 4.73 GHz is complicated, since the SW propagation length is comparable to the SW wavelength, so that the propagation maxima and minima of the dynamical magnetization are not clearly distinguishable. However, it should be noted, that, rather unexpectedly, even for this lowest excitation frequency, that should correspond to the excitation of a ferromagnetic resonance^21^, the propagation of SWs having a non-zero wave vector is seen (Fig. 3(a)). This unexpected feature will be explained below.

Note also, that, as always for the parametric excitation in a continuous media, SW wave number depends only on the pumping frequency and, due to the nonlinear frequency shift, on the pumping amplitude (see below), but not on the length of the excitation gate *L*_*g*_. The propagation length *l*_*p*_ = *v*/Γ of the excited SWs depends on their wave number–it increases with *k*, since the SW group velocity *v* increases with *k* faster than the SW damping rate Γ (until 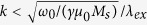, where *ω*_0_ is the ferromagnetic resonance frequency. Otherwise the opposite relation takes place).

From Fig. 3 one can also see that the excited SWs are uniform across the nanowire width. This is absolutely natural, since for such a narrow nanowire all the SW modes that are nonuniform across the nanowire width have much higher frequencies *f*_*SW*_, which simply don’t satisfy the parametric resonance condition *f*_*SW*_ = *f*_*p*_/2. For much wider nanowires (hundreds of nanometers in width) the resonant condition could be satisfied for *several* SW modes having different profiles across the nanowire width. In this case the parametric pumping could excite a nonuniform mode–this depends on the interplay between the SW radiation losses and the coupling with pumping (e.g. see an example in ref. 28).

### Theory

In order to find an explanation to the features of the SW excitation process seen in the numerical modeling and to derive an approximate analytical expression for the SW excitation efficiency we developed a theoretical model presented below. We start from the system of modified Bloembergen equations describing the process of parametric excitation of SWs^29^,^30^:


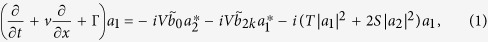


which describes the temporal and spatial evolution of the envelope amplitudes *a*_1_ and *a*_2_ of the excited SWs having the carrier wave vectors *k* and (−*k*) and frequency *ω*_*k*_ = *ω*_*p*_/2, respectively (the second equation of the system could be obtained by the replacement *a*_1,2_ → *a*_2,1_ and *v* → (−*v*)). Here *v* and Γ are the SW group velocity and damping rate (which includes both natural damping and nonuniform resonance line broadening due to technological imperfections), 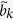 is the Fourier image of the microwave anisotropy field, 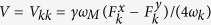 is the efficiency of the parametric interaction, where 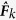 is the Fourier transform of the coordinate-dependent demagnetization tensor^21^. Characteristics of SWs having the carrier wave vectors *k* and (−*k*) are the same, since SW spectrum in the considered perpendicularly magnetized nanowire is reciprocal. Equation (1) contains the so-called nonadiabatic pumping term 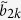
31 which should be taken into account since the SW wavelength is greater than the pumping localization length.

We also take into account the nonlinear frequency shift *T* caused by the SW “self-interaction” and the nonlinear 4-wave (4-magnon) interaction between the parametrically excited “pairs” of SWs having wave vectors *k* and (−*k*), which is described by the Hamiltonian 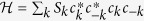, where *c*_*k*_ is the SW amplitude (see “S-theory” in ref. 23). Note, that in bulk samples this 4-magnon interaction between the SW “pairs” is the only important mechanism limiting (or determining) the amplitudes of the parametrically excited SWs. All the other nonlinear interaction processes between the SWs could be disregarded, as in the traditional “S-theory” for bulk magnetic samples (see ref. 23). The validity of this approximation will be confirmed from the comparison between the analytical results of the above *presented* model and the results of the micromagnetic simulations. The magnitudes of the nonlinear coefficients in the model could be evaluated using the formalism of ref. 32 (see Methods). In the range of relatively long SWs (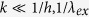) both nonlinear coefficients could be approximately evaluated as 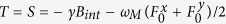, where *B*_*int*_ is the modulus of the static internal field in the nanowire.

The threshold of the parametric excitation of SWs could be calculated from Eq. (1) in the linear approximation, setting *T* = *S* = 0. Following the method from ref. 30 one can obtain an implicit equation determining the parametric excitation threshold:


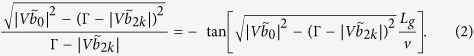


In the limiting case of a small gate length Γ ≪ *v*/*L*_*g*_ one could obtain from Eq. (2) a well-known^31^ explicit expression 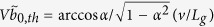, where 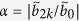 is the measure of the pumping “nonadiabaticity”; in our case of rectangular pumping profile 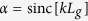. From this expression it is clear that the threshold becomes higher with the increase of the radiation losses Γ_*rad*_ = *v*/*L*_*g*_. Since the SW group velocity *v* is higher for shorter SWs having higher eigenfrequencies, it is clear that the frequency range of the parametric excitation is larger for a larger gate size *L*_*g*_ at a fixed pumping amplitude and, naturally, this range increases with the increase of the pumping amplitude *b*_*p*_. In principle, at a very high *b*_*p*_ the nonlinear processes other than those considered here could take place and could change this tendency. However, this issue lies beyond the scope of our current work.

To calculate the amplitudes of the parametrically excited SWs one needs to find stationary solutions of the system Eq. (1). Note, that at this stage the nonlinear frequency shift *T* could be disregarded, since the SW spectrum is *continuous*, and with the increase of the SW amplitude the SW wave vector changes in such a way, that the resonance condition *ω*_*p*_ = 2*ω*_*k*_(|*a*|) is satisfied. This process, which in not described by the system Eq. (1), will be explicitly discussed below.

The analytical solution of the system Eq. (1) could be found only in the simplest case, when the parametric pumping is spatially uniform and covers all the magnetic film^23^,^30^, while in the general case this system allows only a numerical solution. The analysis of the numerical solutions of Eq. (1) has shown that the amplitude of the parametrically excited SW could be estimated with reasonable accuracy using the following approximate expression:


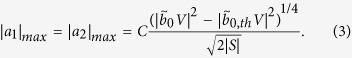


This approximate expression differs from the exact analytical one, obtained for the case of a spatially uniform pumping, only by the presence of the coefficient *C*. Here 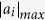 is the maximum value of the SW envelope amplitude in the nanowire, which commonly occurs at the boundary of the pumping area (left and right boundaries for *a*_1_ and *a*_2_, respectively). Thus, it is the maximum amplitude of a SW propagating from the area of pumping localization. The coefficient *C* increases from 1 up to *C* ~ 2 when the radiation losses Γ_*rad*_ = *v*/*L*_*g*_ (existing due to the finite pumping localization and finite SW group velocity) increase in comparison with the intrinsic SW damping Γ. Our simulations showed also that the dependence of the coefficient *C* on the degree of nonadiabaticity *α* is sufficiently weak to be neglected.

The appearance of the coefficient *C* in Eq. (3) is related to the spatial localization of the parametric pumping under the gate of the length *L*_*g*_. In the case of a spatially localized pumping the spatial profiles of the parametrically excited spin waves become nonuniform. Namely, for weakly localized pumping, when Γ*L*_*g*_/*v* ≫ 1, SW profiles *a*_*i*_(*x*) are almost uniform within the pumping region except small regions near the pumping area boundaries. When the pumping becomes more localized (the ratio Γ*L*_*g*_/*v* decreases), these boundary regions occupy larger relative area and finally, for a sufficiently localized pumping, there are no regions with constant SW profiles at all. Nonuniform SW profiles affect both the values of the excitation (*V*) terms and nonlinear (*S*) terms in the right-hand-side part of Eq. (1), an interplay of which determines the excited SW amplitude. For a more localized pumping, when the region where 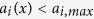 becomes larger, both pumping and nonlinear terms decrease, but the nonlinear *S*-term decreases faster since it contains three spin wave mode amplitudes contrary to one SW amplitude in the *V*-term. Consequently, the resulting maximal spin wave mode amplitude *a*_*max*_ increases when the pumping becomes more spatially localized (for a fixed value of the numerator in Eq. (3)). This effect is taken into account by the coefficient *C* which increases from 1 to roughly 2 with the decrease of the ratio Γ*L*_*g*_/*v*, as was found from numerical simulations of Eq. (1). Note, that this does not mean that a decrease of the gate length *L*_*g*_ leads to an increase of excited SW amplitudes at *given pumping*, because the threshold 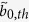, which also determines excited SW amplitudes (Eq. (3)), becomes larger for a more localized pumping, as described by Eq. (2), and the increase of the threshold is significantly greater than the variation of the coefficient *C*.

Let us now discuss the effect of the nonlinear frequency shift (described by the coefficient *T*) on the process of parametric excitation of SWs. As it was pointed earlier, this frequency shift leads to the change of the SW wave vector with the increase of the SW amplitude. If all the parameters in Eq. (1) are only *weakly* dependent on the SW wave vector, one can simply neglect the nonlinear shift in Eq. (1) and use the approximate Eq. (3) for evaluation of amplitudes of the excited SWs. In our case, however, this assumption is not correct. Although the parameters *V*, Γ, and *S* are weak functions of the SW wave vector, the SW group velocity *v* changes significantly with the wave vector *k* variation. Note also, that the nonlinear frequency shift coefficient in our case is negative, *T* < 0, that is related to the perpendicular anisotropy of magnetic nanowire. Thus, with the increase of the SW amplitude the SW spectrum shifts down, and the half of the pumping frequency will now correspond to a higher SW wave vector and, therefore, to a higher value of the SW group velocity. Thus, with the increase of the SW amplitude the radiation losses increase, which leads to the additional limitation of the amplitude of the excited SWs.

To account for this effect rigorously, one needs to insert the dependence *v* = *f*(*k*(|*a*|)) into Eqs (2, 3) and to obtain a complex implicit equation for the excited SW amplitudes. On the other hand, to obtain an approximate analytical solution one can use an approximate dispersion relation for SWs in an *ultrathin* nanowire in the form: 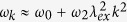, and obtain a simple approximate expression for the SW group velocity in the form: 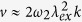. Here *ω*_0_ is the frequency of ferromagnetic resonance and 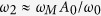 for *k* ≪ 1/*λ*_*ex*_ (see expression for *A*_*k*_, Eq. (9), in the Methods section). The parametric excitation threshold Eq. (2) can be expressed as^23^ 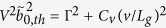, where 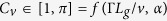. In our case of a highly nonadiabatic pumping and relatively high radiation losses the coefficient *C*_*v*_ is equal to *C*_*v*_ ≈ 1. Combining these expressions and taking into account the amplitude dependence of the SW frequency 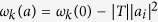 we find that the increase of the SW amplitude leads to the following increase of the total SW losses in the system: 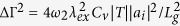. Solving now Eq. (3) taking into account the explicit power dependence of the SW losses, we finally get


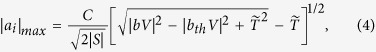


where 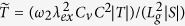. It should be noted, that for a relatively large nonlinear frequency shift 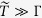, that is realized in our geometry, the resulting SW amplitude, given by Eq. (4), very weakly depends on the value of the coefficient *C*, which cannot be found analytically. Thus, in our case we can simply use *C* = 2, as for the case of a high radiation losses.

Using Eq. (4) we can describe the frequency dependence of the excited SW amplitudes, obtained from the micromagnetic simulations. The quantity calculated from the micromagnetic simulations is the sum of the partial SWs amplitudes at the center of the excitation gate, 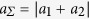. This quantity can be expressed as 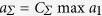, where the coefficient 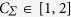. The value *C*_∑_ = 2 corresponds to low radiation losses, when the amplitudes of the forward- and backward-propagating parametrically excited SWs are almost constant within the pumping region. With the increase of the SW group velocity (and, therefore, with the increase of the radiation losses) the value of the coefficient *C*_∑_ decreases. The relation between envelope amplitude *A*_∑_ and the real magnetization amplitude *M*_*x*,*y*_ are given by^23^,^32^


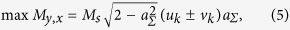


where


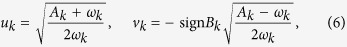


and *A*_*k*_, *B*_*k*_ are the coefficients of the Holstein-Primakoff transformation (see Eqs (9 and 10) in the Methods section).

The resulting analytical expression for the frequency dependence of the amplitude (*m*_*x*_) of the parametrically excited SWs gives a good quantitative description of the results of our micromagnetic simulations for *C*_∑_ = 1.7 (see Fig. 2(a,c)). Note, that *C*_∑_ is the only fitting parameter used, as all the other quantities were calculated from the nanowire geometry and the material parameters. Note also, that if we neglect the effect of the nonlinear frequency shift and use Eq. (3) instead of Eq. (4) the analytically calculated SW amplitudes will be significantly overestimated. Thus, the change of the SW group velocity due to the nonlinear adjustment of the SW wave vector is critically important in the process of parametric excitation of SWs in magnetic nanowires. Also, it is due to this adjustment that we see the propagating SW profiles at the lowest excitation frequency (Fig. 3). The standing ferromagnetic resonance at this frequency could be excited only when the pumping amplitude (and, therefore, the amplitude of the excited variable magnetization) is sufficiently low to produce any evident nonlinear shift of the SW wave vector.

**Figure 4:**
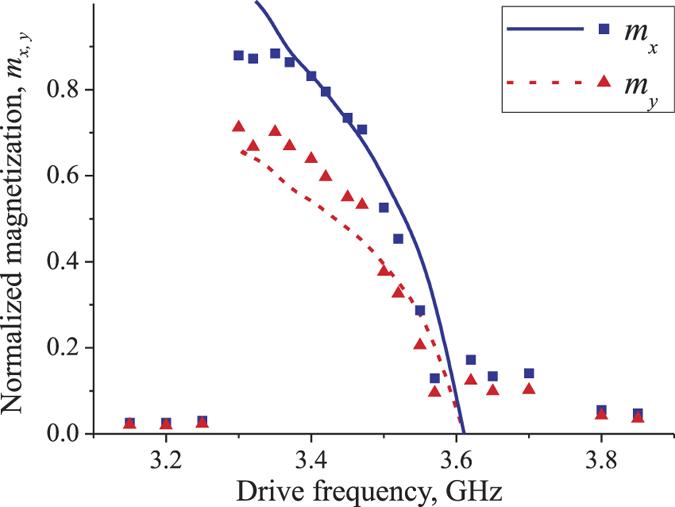
Amplitude of excited spin waves. Normalized in-plane components *m*_*x*,*y*_ = *M*_*x*,*y*_/*M*_*s*_ of the variable nanowire magnetization under the center of the excitation gate as functions of the pumping frequency for Δ*B*_*a*_ = 50 mT, wire width *w*_*y*_ = 50 nm and gate length *L*_*g*_ = 100 nm. Symbols - micromagnetic simulations, lines - analytical theory.

In order to verify the developed analytical theory we performed micromagnetic simulations for a different width of the nanowire (50 nm) and a different gate length (100 nm), setting also the anisotropy constant to 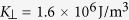. This change did not lead to any qualitative difference in the micromagnetic results, and we observed SW excitation in the frequency range *f*_*p*_ ∈ [3.3, 3.57] GHz, which corresponds to the following wave number range of the excited SWs: *k* ≤ 11 μm^−1^. The developed analytical theory gave a good quantitative description of the micromagnetic results for *C*_∑_ = 1.9 (see Fig. 4). The higher value of the coefficient *C*_∑_ for this case then for the first studied geometry (*C*_∑_ = 1.7) is natural, since in this case the radiation loses Γ_*rad*_ = *v*/*L*_*g*_ are smaller due to larger length *L*_*g*_ of the pumping area.

Finally, let us discuss the issue of stability of the SW parametric excitation. As it follows from the results presented above, the only important nonlinear interactions between the excited SWs are the 4-wave processes responsible for “self-action” (described by the nonlinear coefficient *T*) and the processes of the interaction between the SW “pairs” (described by the nonlinear coefficient *S*). All the other 4-wave scattering processes, which could lead to the SW instability, are weak due to quasi-one-dimensional character of our system (nanowire) and the monotonic character of the of the SW spectrum of the nanowire. The 3-wave processes in our geometry have zero efficiency at all as long as the static magnetization is aligned with one of the symmetry axis of nanowire^32^. The 2-magnon scattering processes, which could take place due to the defects present in a nanowire, in the studied case of ultrathin magnetic nanowires should be weak, since the characteristic size of the possible defects in the nanowire material (nm) is substantially smaller than the characteristic SW wavelength (100 nm and more). Finally, the non-adiabatic character of the applied parametric pumping fixes not only the sum of phases of the excited SWs, but also the *difference* of these phases^31^, which makes the SW excitation process stable with respect to the appearance of magnetization auto-oscillations^23^.

## Discussion

In this work it has been demonstrated that a local microwave variation of the magnetic anisotropy caused by the application of a microwave electric field could excite propagating SWs in an ultrathin ferromagnetic nanowire via the parametric excitation mechanism. The excited SWs could propagate for distances that are large compared to the size of the excitation gate, could have relatively large amplitudes and narrower spectral linewidth comparing to linearly excited SWs. These properties are very desirable for applications of the excites SWs in the nanoscale microwave signal processing devices. It is confirmed that, similar the case of a spatially uniform parametric pumping, the amplitudes of the excited SWs are limited by the “phase mechanism” resulting from 4-wave interaction between the excited SW “pairs”. However, in the case of a spatially localized parametric pumping in a magnetic nanowire due to a significant dependence of the SW group velocity on the wave vector a nonlinear shift of the SW frequency should be taken into account to obtain a correct estimation of the amplitude of the excites SW.

## Methods

### Micromagnetic simulations

Our micromagnetic simulations were performed using the parallel micromagnetic solver GPMagnet^33^,^34^. The length of the nanowire *L* = 2 μm was chosen to be sufficiently large to avoid the possible reflections from the far edges of the waveguide. The gate region of the length *L*_*g*_ was placed at the nanowire center. The material parameters of the ferromagnetic waveguide used in our simulations were: saturation magnetization *M*_*s*_ = 1.6 × 10^6^ A/m, exchange constant *A*_*ex*_ = 2.0 × 10^−11^ J/m, out-of-plane anisotropy constant *K*_⊥_ = 1.55 × 10^6^ J/m^3^, and Gilbert damping constant *α*_*G*_ = 0.01, that corresponds to the typical parameters of CoFeB. Within the excitation gate region the anisotropy field was considered to be time varying: 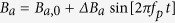, where 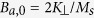 is the static part, whereas *f*_*p*_ and Δ*Β*_a_ are the frequency and the amplitude of the microwave-frequency anisotropy field, respectively. Thermal fluctuations corresponding to the temperature *T* = 1 K were taken into account.

### Calculation of nonlinear coefficients

A general way to calculate the coefficients of nonlinear SW interaction in ferromagnetic films is presented in ref. 32. In the notation of ref. 32 the coefficients *T* and *S* are the 4-magnon scattering coefficients *T* ≡ *W*_*kk*,*kk*_ and 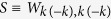. Since the tensor 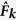 is diagonal (in coordinate system shown in Fig. 1) all 3-magnon processes have zero efficiency until static magnetization is aligned with one of the symmetry axis of nanowire, so one doesn’t need take into account the renormalization of 4-magnon coefficients due to nonresonant 3-magnon processes. Noting this one can obtain following expressions for the nonlinear coefficients in the case of zero external magnetic field


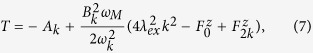



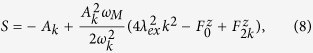


where


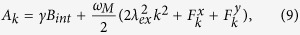



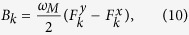



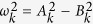
 is the SW eigenfrequency, *λ*_*ex*_ is the material exchange length and 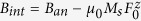 is the static internal field in nanowire. In the range of relatively long SWs (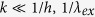) the above expressions reduce to 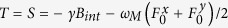.

## Additional Information

**How to cite this article**: Verba, R. *et al.* Excitation of propagating spin waves in ferromagnetic nanowires by microwave voltage-controlled magnetic anisotropy. *Sci. Rep.* **6**, 25018; doi: 10.1038/srep25018 (2016).
